# The lived experiences of street children in Durban, South Africa: Violence, substance use, and resilience

**DOI:** 10.3402/qhw.v11.30302

**Published:** 2016-06-09

**Authors:** Frances Hills, Anna Meyer-Weitz, Kwaku Oppong Asante

**Affiliations:** Discipline of Psychology, School of Applied Human Sciences, University of KwaZulu-Natal, Durban, South Africa

**Keywords:** Lived experiences, resilience, street children, substance use, violence, South Africa

## Abstract

South African studies have suggested that street children are resilient but also suicidal, engage in unprotected sex and other high risk sexual behaviour as a means of survival, have high rates of substance abuse and are physically abused and stigmatized due to their state of homelessness. However, few studies have explored in a more holistic manner the lived experiences of street children in South Africa. The main purpose of this study was to explore qualitatively the lived experiences of street children living on the street of Durban, in the province of KwaZulu-Natal, South Africa. Adolescents (six males and four females) between the ages of 14 and 18 years (average age=16) were purposively selected and in-depth semi-structured interviews were conducted. An interpretative phenomenological analysis of the transcribed data revealed that incidence of violence and drug and alcohol use were common experiences of street life. Yet despite these challenges survival was made possible through personal and emotional strength, cultural values, religious beliefs, supportive peer relationships, and participation in sports activities. These protective, resilience resources should be strengthened in health promotion interventions with a focus on mental health, the prevention of violence, substance use, and daily physical activities that seems to provide meaning and hope.

“All those who pass by see them, but they are invisible. They do not exist.” This is how researcher Elena Poniatowska describes homeless youth on the streets of Mexico City in the United Nations International Children's Emergency Fund (UNICEF, 2006) report on the *State of the World's Children 2006: Excluded and Invisible* (p. 42). It is, however, not only Mexico City's homeless youth who are invisible. The same might be said of those in South Africa, in whose cities homeless children and youth have been estimated to number in the thousands (Human Sciences Research Council [HSRC], 2008; Sewpaul, Osthus, Mhone, Sibilo, & Mbhele, 2012).

The United Nations Education Fund (UNICEF, 2015) has estimated that there are tens of millions of street children and adolescents globally in both developed and developing countries. In South Africa, about 250,000 children and adolescents are reported to be living on the street (Consortium for Street Children [CSC], 2014). Most are in the large cities, including Durban, the second largest city in South Africa (Statistics South Africa [StatSA], 2014). The inherent difficulties in counting homeless youth due to their mobility were noted by Ward and Seager (2010) when working on the HSRC's 2005–2008 large-scale study of the homeless in the Gauteng and Cape provinces of South Africa. Nevertheless, the large number of children living on the streets provides a challenge to government, policymakers and non-governmental organizations.

Most of South Africa's homeless youth are black and male (HSRC, 2008), with the gender disparity attributed to the widespread practice of girl children being tasked with home responsibilities such as child-minding and other household chores (Le Roux, 1996). Youth in South Africa also seem to stay on the street longer than those in developed countries. The HSRC survey (2008) found that the majority of homeless people (adults and children) surveyed had been on the streets between 2 and 5 years, with some for far longer. Duration on the street is likely to impact full reintegration into society.

Experiences of parental neglect, physical and sexual abuse, and extreme poverty are common drivers to a life on the street (Oppong Asante, 2016; Oppong Asante & Meyer-Weitz, 2015a; Seager & Tamasane, 2010; Ward & Seager, 2010. This in turn increases their vulnerability to both health risk behaviours and mental health problems, which is further exacerbated by limited access to education, support, and health facilities.

The resilience of street children has been highlighted in South African studies (Theron et al. 2011; Theron & Malindi, 2010). However, they are also suicidal (Ward & Seager, 2010), engage in unprotected sex and other high-risk sexual behaviours as a means of survival (Kruger & Richter, 2003; Van Rooyen & Hartell, 2006), have a high rate of substance abuse (Morojele, Parry, Brook, & Kekwaletswe, 2012), and are physically abused and stigmatized due to their state of homelessness (Ogunkan & Adeboyejo, 2014; Oppong Asante, Meyer-Weitz, & Petersen, 2015). All these aspects also increase their risk for HIV infection, especially in the context of a generalized HIV epidemic. Furthermore, street children have also been reported to have poor mental health partly due to challenging lifestyles (Aptekar & Stoecklin, 2014a; Swart-Kruger & Richter, 1997; Van Rooyen & Hartell, 2006; Ward & Seager, 2010). Notwithstanding these studies, a deeper exploration of the lived experiences of street children in Durban, South Africa, is needed as to the type of violence they are exposed to or the survival strategies they adopt to overcome challenges on the street.

The main purpose of this study was to qualitatively explore the lived experiences of youth living on the street of Durban, a coastal city in KwaZulu-Natal, South Africa. It is envisaged that the findings of this study could inform the development of interventions to address the needs of this vulnerable population.

## Theoretical frameworks

The resilience theory of Ungar (2008, 2011) and the risk and protective factor model of Hawkins, Catalano, and Miller (1992) were used as frameworks to explore the lived experiences of street youth. Resilience theory is generally concerned with the ability of an individual to overcome, positively adapt, or cope with adversity (Masten, 2001). It stems from the perspective that numerous factors both within and outside of the individual or child combine to determine the general course of development as well as specific behavioural patterns (Ungar, 2011). Over the past two and half decades, several explanations have been postulated to explain the complex nature of resilience, but only three different types of models have been described. A more recent ecologically focused definition of *resilience* was provided by Ungar (2008), who stated that “in the context of exposure to significant adversity, whether psychological, environmental, or both, resilience is both the capacity of individuals to navigate their way to health-sustaining resources, including opportunities to experience feelings of well-being, and a condition of the individual family, community and culture to provide these health resources and experiences in culturally meaningful ways” (Ungar, 2008, p. 225). Thus, in understanding how street youth survive in the adverse environment in which they find themselves, and how they make meaning of their experiences, it is appropriate to consider individual-level influences but also the interaction between, and within, the social and environmental context in which the individual lives (Ungar, 2011).

The risk and protective factors model (Hawkins et al., 1992) outlines factors within a particular population that may ameliorate the effects of psychological problems and or distress (protective factors) or exacerbate the probability of developing a psychological problem (risk factors). Among vulnerable populations, several factors may be associated with susceptibility to various health risk behaviours. These include personal and situational characteristics that may directly impact mental and physical health, or indirectly *via* acceptable and accessible health services, social resources, and support. Certain sociodemographic characteristics, for example, their younger age and lack of adult guidance, years of living on the street, and lack of education, make them particularly vulnerable to engagement in health-compromising street lifestyles. These lifestyles may include poor health-seeking behaviour, vulnerability to physical and sexual abuse, and maladaptive coping strategies, including the use of drugs and alcohol that may have major long-term effects on the mental health of street children. This further underscores the need for a deeper understanding of risk and protective factors that are associated with homeless youth's street lifestyles to support appropriate harm-reduction interventions and increased access to appropriate psychosocial and health services.

## Methods

### Research design

The study adopted a qualitative research design, which enables researchers to capture how those being interviewed view their world, to learn their everyday language and judgments, and to capture the complexities of their perceptions and experiences (Neuman, 2011). It also helped to give the authors an in-depth understanding of the youths’ subjective understandings and experiences of homelessness.

### Sampling and participants

The non-probability sampling technique of purposive sampling was used to select research participants from a drop-in centre in Durban. Participants were recruited for the study if they were homeless, had lived on the street for a month or longer, agreed to participate in the study, and were willing to answer questions related to their life as lived on the street. The sample consisted of 10 adolescents (six males and four females) between the ages of 14 and 18 years (average age=16). Participants’ length of time being homeless varied from 2 months to 12 years. Eight of the participants identified themselves as black South Africans, while two were coloured (of mixed race). Apart from one, nine of the participants had a basic education, with one not remembering the level of education.

### Data collection and procedure

Ethical approval for the study was obtained from the Human and Social Science Ethics Committee of the University of KwaZulu-Natal, South Africa (protocol number: HSS/0958/012) and the administrator of the drop-in centre. Two research assistants (male and female) helped to conduct the interviews in IsiZulu at the drop-in centre, which provides psychosocial services to street children in Durban. The aim of the study was explained, and participants were assured of confidentiality and voluntary participation. Participants were informed of the availability of a psychologist should they require such a service. Those who agreed to participate in the study signed an informed consent form. Permission to audio-record the interviews was also sought from each participant. Interviews were then conducted in the preferred language of the participants and at a place convenient to them. An interview schedule developed from a review of both international and South African literature was used as a guide, allowing for further probing where appropriate. The interview schedule was developed in English and translated into IsiZulu and then back-translated into English to ensure consistency for the key questions. Some of the interview questions asked were: *How do you cope with conditions on the street? What do other people do to cope with bad things that happen to them on the street? What kind of resources/structures do you rely on in the streets? Do you think religion has a role to play in your life?* The interview schedule only served as a guide, and probing questions were used to explore the youths’ own views and experiences during the interview process. Eight participants were interviewed in IsiZulu while the remaining two were interviewed in English. Interviews took approximately an hour to complete.

### Analysis

All audio-recorded data were transcribed and translated into English. Data were thematically analysed using the four steps of interpretative phenomenological analysis (Storey, 2007). Coding of the data was informed by *a priori* concepts (Storey, 2007) as presented in the interview schedule and new emergent themes from their lived experiences as reflected in the interviews. In the first step, the authors read and re-read the transcribed data to familiarize themselves with the data and made notes. The second step involved harmonizing, identifying, and labelling themes from the notes that had been identified. In the third stage, these themes were connected to develop a main theme. In the final analysis, we summarized the main themes with their subthemes, with supporting illustrative quotations before the final narrative form. Two independent coders were engaged to cross-validate the emergent themes. The NVivo software (version 10.1)(QSR International, Boston, USA) was used in generating codes from the data (some of which were pre-identified using the interview guide) and organizing these codes into various themes. The themes, which were identified by the first author, were cross-validated by the second and third authors in order to reduce subjectivity and increase the validity of the identified themes.

## Results and discussion

Following the analysis of the data, the findings were organized into three major themes: (a) violence as an everyday experience, (b) substance use as coping, and (c) psychosocial and contextual resources of resilience.

### Violence as an everyday experience

Notions of living on the streets and being homeless are intertwined with experiencing and witnessing aggression and violence. While the interviewers did not raise the issue of violence, the focus was on understanding how street youth cope with daily living. It was thus inevitable that the issue of violence was often spoken about. Three sub-themes around violence emerged, namely rape, violence, harassment and other acts of aggression.

Females were often the victims of rape on the street. Some respondents recounted how girls live in constant fear because of “what happened to a friend.” For example, one girl said:

Recently, my friend got stabbed in her private parts to death by a group of guys that wanted to rape her and she tried to fight them. She died three days after the incident. My friend died a slow and painful death. This incident was hard to deal with because I'm also a girl and therefore I am a potential target for rape as well. (Participant 1)

Another female interviewee poignantly disclosed that she, herself, had been raped:

There are boys that rape us, who don't see us as sisters, but as girls that are there to get raped. I myself was raped by a guy. When it happened I felt so violated and alone. I felt so powerless when I got raped I did not even try to fight back. I just let him do whatever he was doing and I just kept on crying. (Participant 3)

These accounts by the females are an indication of how overwhelmed and powerless they are on the street. It seems that rape is a common occurrence on the streets of Durban, at least for females. Indeed, in an earlier study, one of the female participants in Motala and Smith's (2003) study reported having been raped three times in her 4 years on the streets of Durban. Previous findings have indicated that sexual assault is a common occurrence on the streets, as young boys are also sexually assaulted by older boys (Seager & Tamasane, 2010). Male participants in our study did not mention sexual assault; this was in contrast to an earlier study that found widespread fear of rape among both female and male participants, some of whom had already been raped (Motala & Smith, 2003). In fact, the boys interviewed in the Motala and Smith's (2003) study disclosed they slept in different places each night to avoid being raped. Our findings also support a previous study where female street children were found to be victims of male violence as a means of controlling their sexuality, subjugating them to masculine dominance, and keeping other boys away from them (Sewpaul et al., 2012).

It is not very surprising that rape is so common among street youths. South Africa is reported to have one of the highest statistics of violence against women in the world (Dartnall & Jewkes, 2013; Jewkes, Flood, & Lang, 2015), and the fact that South Africa marks 16 days of action against violence against women every year shows that levels of violence directed at women are of national concern. The lack of protection and shelter puts homeless youth at a greater risk for rape than the general population of South Africa, whose women, in particular, are at a high risk of being raped (Jewkes et al., 2015).

Violence and physical harassment from the police were another form of violence that male street children in particular experienced. For example, two respondents said that the homeless males, in particular, are badly treated by this city's police force. One of the males said: “Metro Police abuse people living on the street and beat them,” whereas the other reiterated that, “At night, the problem is the Metro Police. They kick us, beat us and stomp on us as if they are superior human beings than us.” These findings with regard to the abuse from the police echo those of Sewpaul et al. (2012), who conducted focus group and individual interviews with Durban street youth over a period of 2 years. They described the accounts of violence and abuse at the hands of the Metro Police they heard from participants as “horrific” (p. 247), saying that such reports also formed a common theme in their research. In fact, these researchers were forced to start a focus group late one day because the participants arrived late and exhausted after walking 74 kilometres from Stanger, far north of Durban, where they said the Metro Police had dumped them after beating them up the night before.

A previous study conducted in South Africa also described harassment and assault at the hands of both the Metro Police and the South African Police Services (Seager & Tamasane, 2010). These youngsters also said that whenever a criminal act was discovered, they were the first to be suspected by the police and were chased away from buildings in the Johannesburg Central Business District. A qualitative study with Durban street children in 2002 found that most of the children interviewed said that the individuals they feared most on the street were the police (Motala & Smith, 2003). Donald and Swart-Kruger (1994) found that harassment and arrest by the police were common and claimed that the lives of street children amounted to a “constant cycle of arrest, release or escape from arrest and re-arrest” (Swart-Kruger & Donald, 1994, p. 109). The participants in the current study did not mention actually being arrested but spoke of harassment, being taken far away from the streets and dropped without transport, and of violence from the Durban Metro Police.

Violence and harassment at the hands of the police is not confined to South Africa. Other studies conducted in Nepal and Zimbabwe have reported that street children are frequently harassed by the police, who in some cases demand bribes from street youth in exchange for ceasing to harass the children (Baker, Panter-Brick, & Todd, 1997). These types of treatment at the hands of the police violate a number of children's rights as laid out in the South African Constitution, the United Nations Convention on the Rights of the Child, and the legislation and policies drawn up to protect children that operate at regional and national levels in the country (e.g., Panter-Brick, 2004; Sewpaul et al., 2012).

Other forms of violence, such as fighting, were also mentioned by participants. For example, one male said that he had been beaten up and otherwise ill-treated by boys who had been on the streets longer than he had. He continued as follows:

Since I have arrived in the streets, there are so many people I know who have died on the streets, stabbing and killing each other. You know—things like that. (Participant 3)

The allegations against older street males confirm earlier research among Durban street youth who reported being beaten by older boys on the street, gangs, homeless adults, security guards, and, as mentioned above, the police (Motala & Smith, 2003).

A male participant confessed that he, too, resorted to violence once he realized that no one cared about anyone on the streets. Another male reported a similar story:

Especially when I first arrived, they [older boys] used to abuse me, beating me up, demanding my money, giving me “blue eyes.” But I also ended up fighting back after having had enough. (Participant 10)

These findings correspond with those of Seager and Tamasane (2010), who found that homeless people, in general, are very prone to assault and injury. They found strong evidence of drug and alcohol-related violence, beatings, and robberies carried out by older boys on younger boys, gang-related violence, and assaults by members of the public. These violent behaviours concurred with what Sewpaul et al. (2012) found in their Durban study, namely that violence was endemic, with most of their survey respondents witnessing violence several times a day. The researchers emphasized the importance of paying attention to the issue of control when considering violence among street youth. They argued that violence could be seen as a strategy towards exerting control over others, such as older boys over younger boys. Violence on the street is generally considered the norm and determines the ability to survive on the street (Oppong Asante, 2015, 2016).

### Substance use as coping

The use of drugs and alcohol has been found to be widespread among homeless youth. Even though participants were not asked about their or others’ use of drugs or alcohol, several spoke spontaneously about these practices when discussing how they deal with adversity. For example, one girl said:

To cope, I smoke cigarettes because I think it helps me to cope with the stress of living on the streets. I and my friends love drinking, smoking, and having fun at the clubs. (Participant 1)

The behaviour expressed by this participant seemed to derive pleasure from the freedom they have on the street. The desire to be free from parental control is an important reason for youngsters to leave home to live on the streets (Oppong Asante, 2016; Ursin, 2011). The sense of freedom that the girls from the current study found in partying, drinking alcohol, and smoking cigarettes may have served as form of escapism from harsh conditions on the street. Indeed, Scanlon et al. (1998) reported that around 80% of street children in Latin America utilized drugs on a regular basis as a cheap way of coping with hunger, fear, loneliness, and despondency. This also corroborates a South African study in which “smoking glue” generated pleasant feelings, as well as shutting out loneliness, hunger, cold, and insecurity (Donald & Swart-Kruger, 1994).

A male participant spoke of sniffing glue (which provides a cheap and easy-to-access “high”) and how he perceived it to help him by allowing him to escape from the stressors on the street:

You smoke glue if you don't want to keep thinking about your situation, because when you smoke it, you get high and you hallucinate; you don't have to keep thinking that you live on the streets and all that stuff. I don't blame anyone who snorts glue, because glue takes away the sadness of living in the streets; plus glue is helpful in that it prevents a person from doing more dangerous and heavier drugs like Whoonga, cocaine, and ecstasy. Heavy drugs are bad; I have tried them and I have seen other people vomit and cough up blood and be sick because of them. (Participant 1)

Another participant revealed that smoking glue made things “difficult” for him and described what life was like for a drug-addicted street youth:

It makes me physically sick. You also crave it when you don't have money for it, but you still have to find some way to buy it. And then you end up not having any money to buy food and get high on weed [marijuana], and then when the high is over, you are very hungry, and you don't have food, and your money is gone because you spent it on glue. I try to only smoke weed and cigarettes. (Participant 7)

The above narratives indicate that the lifestyles of these participants appear to revolve around drugs. According to criteria set out in the Diagnostic and Statistical Manual IV-TR (Sue, Sue, & Sue, 2006), what this youngster said revealed symptoms of a substance-related disorder. For example, he knew that smoking/sniffing glue was not good for him, but he seemed unable to cut down or control his use, and he devoted a considerable amount of time to activities that allowed him to obtain glue. Other symptoms necessary for a clinical diagnosis might be in place but were not revealed during the interview. He was also in danger of violence or arrest by the authorities as a result of his drug-use behaviours.

Another participant narrated a violent incident that took place after glue-sniffing:

One day we [a friend and I] had a fight after we were smoking glue. After that, we fought over the actual glue bottle. We fought and he took out a knife and stabbed me, but I blocked it with my arm and I got stabbed on my forearm. Then I took a brick and hit him on the head and made him bleed because he had made me bleed too. (Participant 10)

One of the girls showed how normalized drug use was and how it formed an integral part of her life on the streets:

In the morning, I wake up, open my eyes, take a bath, brush my teeth, eat, go pick weed, come back, find a corner with my friends, and smoke weed. (Participant 4)

When this female participant spoke of picking and smoking marijuana in the same sentence as getting up and brushing her teeth, she was portraying drug use as part of daily life. Marijuana smoking also appears to act as a social bond between this participant and the friends with whom she smokes it. In a rather unconventional way—communal marijuana smoking—this participant demonstrated bonds with her peers, a source of resilience (Malindi & Theron, 2010) but possibly what could only be dubbed as a negative coping mechanism.

Our findings confirm the findings of previous studies that revealed the use of intoxicants such as glue, petrol, and benzene to be widespread in their review of the international literature pertaining to homeless youth (Aptekar & Stoecklin, 2014b; Motala & Smith, 2003). The cheap and easily obtainable nature of “glue” allows all ages of children and youth to use it. Oppong Asante, Meyer-Weitz, and Petersen (2014) and Young (2003) reported on the use of fuel among street youth in Uganda and Ghana, respectively.

The findings of the current study also confirm those of the HSRC (Seager & Tamasane, 2010), who showed how common drug use is among South African homeless youth. According to this research, it is commonplace for the homeless to exhibit both direct and indirect symptoms of substance use. These researchers reported various direct consequences of substance use, such as liver disease, as well as indirect consequences such as risky sexual behaviour resulting in a high prevalence of sexually transmitted infections, including HIV.

### Psychosocial and contextual protective factors of resilience

Even though the lifestyles of street youth seem to revolve around incidences of violence and drug and alcohol use, they have found ways of surviving these challenging conditions on the street. The fact that some of these young people have lived on the streets for years points to adaptability and to a strong ability to cope with adversity and thus the harsh circumstances of street life. Although street children face developmental risks in various developmental domains, evidence point to their ability to cope and adapt to extremely difficult circumstances (Ungar, 2011). The protective factors that were identified by participants in this study as helping them cope with the adversity on the street were personal and emotional strength, culture values, religious beliefs, supportive peer relationships, and participation in sports activities.

#### Personal and emotional strength

Personal and emotional strength were emphasized by participants as essential for survival on the street. For them, this mostly seems to entail a physical ability rather than an internal quality and shows some pride in their physical prowess. This physical strength is important for survival on the streets. As one female participant implied, without at least some physical strength, most of these youngsters could easily become victims of violence. As reiterated by a participant, “Everyone fights to live.” What this suggests is that to lack physical strength, then, seems to mean greater vulnerability to the very real physical dangers that come with life on the streets. It also seems that these youngsters not only require physical strength, but also need to physically demonstrate that strength. This possibility arose when considering the words of a female participant who seemed to feel a sense of failure because *she did not use physical force against her rapist*. In spite of her horrific experience, however, this young woman is still resilient enough to survive on the streets and dream of becoming a chartered accountant one day.

Another female participant indicated that she was strong and able to survive street life because she is able to stand up for herself. She also expressed pride in her ability to control herself and not be angry for too long:

I try to control and contain myself in all situations so that I can cope and survive. I try to be calm. The problem is that I easily get irritated by people and I don't like talking too much; sometimes when I am pressured or provoked I end up fighting with people. (Participant 7)

This participant shows how physical strength can sometimes get out of hand when she speaks of her difficulty in controlling her anger and impulse to fight. This girl appears to have some difficulty with impulse control. The same participant, however, also expressed pride in managing to refrain from acting violently and was clearly conscious of what she expressed as a need to control herself and “remain calm in all situations.” This girl spoke of other incidents of physical fighting in her history, a problem that constituted a prominent reason for why she ended up on the streets.

Some of the respondents conceptualized “strength” as something other than physical strength, but as more of an inner quality, which was referred to as “emotional strength.” For example, one of the male interviewees said he saw himself as a strong person:

You see, there's a difference between a kid from the township and a street kid. In the street, there is no one to coddle you whenever you're sick or have [the] flu and stuff like that. (Participant 8)

This participant is clearly referring to the fact that he has no adult caregiver to act with his welfare in mind; there is no buffer between him and harsh reality, and he has been forced to grow up before nature intended. This inner strength echoes what has been described as personal strengths by Theron and Malindi (2010). In fact, they found strong evidence of what they termed “feistiness and stoic staying-power” (p. 727) as contributing to resilience among the homeless young people in South Africa.

An interesting response to the question about strength came from a male participant. He said he considered himself to be a strong person because he was “still hopeful.” This participant seemed to value a personal characteristic that could be described as an open and accepting attitude, and he seemed to consider this to be a form of strength. Hope, a key construct of psychological capital, is an important intrapersonal resource to draw from in times of adversity (Okafor, 2015). Interestingly, some studies (e.g., Oppong Asante & Meyer-Weitz, 2015b; Rew, Taylor-Seehafer, Thomas, & Yockey, 2001) have found that youth who perceive themselves as being resilient report lower levels of life-threatening behaviours, loneliness, and hopelessness. For the participants in this study, their experiences on the streets were perceived to make them tough and capable to live on the streets. This insightful response shows strength of character and also demonstrates an ability to overcome tough experiences and to survive the harshness of life on the streets.

#### Cultural values

The role of cultural values in survival on the street were explored among the participants, as it has been suggested that cultural values are an important source of resilience (Oppong Asante, 2015; Theron et al., 2011; Theron & Malindi, 2010; Ungar, 2011). However, this issue received surprisingly limited responses from most interviewees despite extensive probing regarding, for example, the use of rituals, cultural norms, the connection to ancestors, and wise words of relatives, and so on in encouraging participant responsiveness. Many respondents focused their attention on whether or not they “believed” in the ancestors, in religion, or in intermediaries between humans and a god.

Another young male echoed these sentiments:

No, no, I just believe in God only, not ancestors … I grew up knowing that a witchdoctor does not go to heaven … God is the person who created me, and I see that this is his will that he is doing with me, so I just leave everything to Him. Culture and related rituals do not help me during difficult situations. Like I said, my friend, I don't do anything that has to do with ancestors or cultural rituals. I trust in God only. (Participant 10)

In contrast to the above quote, a female participant revealed that she placed her faith in the ancestors and credited them with her protection on the street:

My ancestors have helped to protect me during some difficult situations in my life. When there are fights here in the streets where I am involved, somehow I never get stabbed, nor am I taken to jail like my friends and brothers. This is one of the reasons that make me believe only in my ancestors. (Participant 7)

In contrast to Theron and Malindi's (2010) study, where they found a preponderance of accounts of resilience-inducing cultural pride, participants in the current study said very little about their culture. In fact, when asked about their ethnicity, all the black youth, except for one, identified themselves as black rather than Zulu or Xhosa. The majority of the participants dwelled upon the issue of whether or not they “believed” in the ancestors. It was almost as if these youngsters had shed their cultural “mantle” as a way of rejecting their origins and accepted the “culture” of street life instead. It is possible that the mentioning of the ancestors in the question about culture proved too provocative for participants; it is possible that in their efforts to distance themselves from “beliefs” in the ancestors, they ended up distancing themselves from their entire cultures, along with their norms, teachings, and practices. It is important to note that participants in Theron and Malindi's (2010) study were unable to articulate how culture had encouraged their resilience. Perhaps this problem resided with our study participants as well. Both Theron and Malindi's (2010) research and the current research have highlighted the need for further research into culture as a resource for resilience.

#### Religious beliefs

In contrast to responses about culture in general, religion (which can be said to constitute a specific element of culture) appeared to play an important role in the respondents’ lives and in their ability to tolerate the difficulties they experienced as street-dwellers. Although the majority of the participants professed to be Christians, some believed in their ancestors. One female participant showed her strong belief in God and attributed her survival on the street to him:

I have seen God play a major role in my and my family's life because I see him protecting me from a lot of things. I pray every morning and I thank God every time for all the things he does for me … my religion plays a major role because I think that God protects me from a lot of bad things that I am facing here on the streets, living and surviving. (Participant 4)

In reference to the hardships involved in street life, one boy said he placed his hope in one “person,” God:

God is the person who created me, and I see that this is His will that He is doing with me, so I just leave everything to Him … He has kept me alive while living in the streets all this time; I could [have been] stabbed or beaten up by people and died, but I'm still alive. That's how I know that God exists and answers my prayers. (Participant 5)

Our findings support previous studies conducted in Ghana and South Africa that found religion to be a major source of resilience for young street children, mostly as a way of making them feel strong, thus making prayer to God fundamental to their hardiness (Malindi & Theron, 2010; Oppong Asante, 2015). They also said that religion was something not typically associated with street children, but that it was a major resource in these youngsters’ ability to rebound from adversity. Participants in the current study echoed the findings of Malindi and Theron (2010), where homeless youth expressed a belief in a higher power that led them to access beneficial outcomes from individuals or communities.

#### Supportive peer relationships

Social relationships and peer support were found to contribute to resiliency in street children in this study, as they often referred to important supportive peer relationships. The majority of the participants seemed to have benefited from the help of a kind friend who was ready to share and help them when in need. One particular older girl featured largely in the lives of several of the female respondents. This girl, to whom the fictitious name of Bess has been given, seemed to have provided a great deal of help to the girls. As a female participant indicated:

When I first got here [on the streets], my friend [Bess] whom I got on the street used to hustle for me and she would also give me some of her clothing … she helps me a lot here on the street because she knows a lot of things. When I am about to get into a fight, she is there immediately to break up the fight before it starts. (Participant 1)

Another girl said that Bess had been a big help at the start of her life on the streets:

When I first got here, [Bess] used to help me a lot because I'd get everything from her from the money she would get from selling CDs to people on the streets. (Participant 3)

It appears that these girls had been “shown the ropes” by Bess, who seemed to act as peacemaker, guide, protector, advisor, and what has been described as a “local role model” by some researchers in Africa (Theron & Malindi, 2010). Indeed, Malindi (2014) found that street youth often adopt one another or other street people as role models. The help offered from an older homeless female in this study contrasts sharply with the treatment respondents reported receiving from groups of older street boys, who were reported to behave in a violent and threatening manner towards the respondents, both male and female. In contrast, a Zimbabwean study (Bourdillon, 1994) found that very often, once street boys got older, they began to act as guardian to a group of younger boys, helping them with money, food, and, crucially, protection.

Research into friendship among children living on the streets has shown that a strong ethos of support between friends constitutes a survival strategy (Malindi, 2014; Mizen & Ofosu-Kusi, 2010). In South Africa, Malindi (2014) found that when street children band together, they form a grouping that provides both emotional and economic support. In support of this, Malindi and Theron (2010) revealed that bonding between street children acts as a strong contributor to the resilience of these youngsters. Contrary to this, Swart-Kruger and Donald (1994) argued that peer groups on the street are often depicted as “quasi-families” because of the support, companionship, and protection that they afford members, but that this is a misleading analogy. According to these authors, the relationships within the street group are more erratic and temporary than within the family group and operate at a very different level from relationships between adults and children in a family. Indeed, research undertaken in Ethiopia (Aptekar & Heinonen, 2003) found street groups to be loose-knit as a result of the children's strong desire for autonomy, which, the researchers claim, actually prevents bonding. According to Swart-Kruger and Donald (1994), the peer group is still important. According to Mizen and Ofosu-Kusi (2010), terms such as *reciprocity*, *cooperation*, and *mutuality* are largely absent from writings about street children, and they point to the general lack of research into friendship among street children. Although South African researchers (e.g., Le Roux & Smith, 1998a; Theron & Malindi, 2010) have paid some attention to this matter, there still seems to be room for further investigation into this area.

#### Participation in sports activities

Community-based support and services provided by drop-in centres seem to be important resilience resources to street youth. Without prompting, the majority of the respondents identified surfing as a physical activity that helped them to cope and gave meaning to their lives. One of the female participants indicated how surfing helped her in daily life and how she hoped to make a meaningful life from it.

Surfing also plays a major role in me being able to cope. When I am surfing, I am free and I am not in anyone's business. If I get surfing sponsorship, things could change for the better for me, if only I could get a surfing sponsorship. The sponsorship will give me proper new clothes, all the surfing equipment I need for competitions, a monthly allowance, and other things as well. All that I need now is to get a sponsor that would help me achieve my dreams of going worldwide to surfing competitions. Through the sponsorship, I could also get my own flat and not have to live on the streets. (Participant 3)

The above participant's determination and the clarity of the picture she had of her future as a professional surfer was confirmation of this young girl's hopefulness and resilience. One of the boys also expressed his hopes and dreams for the future as follows:

Bro’ [brother], it's definitely being more successful at surfing. We also go to competitions and get cash prizes, but it's only for number one until quarter-finalists, and I have never been a quarter-finalist and I want to do well so I can be better. (Participant 2)

The above participant's narrative shows that his dreams and plans are achievable, and he has the determination to achieve just that. From these narratives, the sports programmes that these youth are exposed to offer them hope for a medium-term solution to their problems (sponsorship, getting prizes, accommodation, and so forth), as well as hope for success in the long term. This corresponds with widespread findings reported by Van Blerk (2011), who stated that engaging “at risk” groups such as street children in sport has been found to divert attention from the negative aspects of their lives. Our findings also contradicts the “live for today” attitude of some street youth in Indonesia (Beazley, 2003), as they expressed hope and dreams for the future.

### Limitations

This study has strengths and limitations. The main strength of this study resides in allowing marginal voices to be heard, one of the first studies to have examined the lived experiences of street children in a more holistic approach. Limitations, however, include the small sample size and the non-probability sampling method used, implying that the findings cannot be generalized to all street children in South Africa. Participants who chose to participate in the study may be different than their peers who did not. Furthermore, social desirability biases may have influenced responses to sensitive questions about violent behaviours and substance use. However, such an effect may have been moderated by the strong rapport the research team had with the participants. Despite these shortcomings, the study provides researchers and policymakers with a holistic picture of the life of street children within a South African context.

## Conclusion

The current study was conducted to explore the lived experience of street children and adolescents in Durban, South Africa. Our findings revealed that incidence of violence and drug and alcohol use were common in these youngsters’ lives. Despite substance use being illegal and a socially unacceptable way of coping, it allows them to escape and “check out” of their lives, at least temporarily. In the midst of these challenges, participants derived ways of survival by relying on personal and emotional strenghts cultural values, religious beliefs, peer relationships and support, and participation in physical activities such as surfing. This study contributes to the body of knowledge regarding the lived experiences of street children in Durban, South Africa. Additionally, the identified protective factors could be strengthened in health promotion interventions with a focus on mental health, prevention of violence, substance use, and daily physical activities that seem to provide meaning and hope.
